# Freeform fabrication of tissue-simulating phantom for potential use of surgical planning in conjoined twins separation surgery

**DOI:** 10.1038/s41598-017-08579-6

**Published:** 2017-09-08

**Authors:** Shuwei Shen, Haili Wang, Yue Xue, Li Yuan, Ximing Zhou, Zuhua Zhao, Erbao Dong, Bin Liu, Wendong Liu, Barrett Cromeens, Brent Adler, Gail Besner, Ronald X. Xu

**Affiliations:** 1 School of Engineering Science, University of Science and Technology of China, Hefei, Anhui 230027 People’s Republic of China; 2 First Affiliated Hospital of Anhui Medical University, Hefei, Anhui 230022 People’s Republic of China; 3 Department of Biomedical Engineering, The Ohio State University, Columbus, Ohio 43210 United States of America; 4 Department of Pediatric Surgery, Nationwide Childrens Hospital, Columbus, Ohio 43205 United States of America; 5 Department of Radiology, Nationwide Childrens Hospital, Columbus, Ohio 43205 United States of America

## Abstract

Preoperative assessment of tissue anatomy and accurate surgical planning is crucial in conjoined twin separation surgery. We developed a new method that combines three-dimensional (3D) printing, assembling, and casting to produce anatomic models of high fidelity for surgical planning. The related anatomic features of the conjoined twins were captured by computed tomography (CT), classified as five organ groups, and reconstructed as five computer models. Among these organ groups, the skeleton was produced by fused deposition modeling (FDM) using acrylonitrile-butadiene-styrene. For the other four organ groups, shell molds were prepared by FDM and cast with silica gel to simulate soft tissues, with contrast enhancement pigments added to simulate different CT and visual contrasts. The produced models were assembled, positioned firmly within a 3D printed shell mold simulating the skin boundary, and cast with transparent silica gel. The produced phantom was subject to further CT scan in comparison with that of the patient data for fidelity evaluation. Further data analysis showed that the produced model reassembled the geometric features of the original CT data with an overall mean deviation of less than 2 mm, indicating the clinical potential to use this method for surgical planning in conjoined twin separation surgery.

## Introduction

Conjoined twinning is a rare congenital malformation^1–5^. Although most of the conjoined twins succumb in utero or are stillborn at birth, approximately 1: 30,000–1: 200,000 are born alive. These conjoined twins often display life-threatening conditions and severe disabilities, posing significant challenges to both healthcare providers and patients^6^. Surgical separation is possible in ~1 out of 650–900 conjoined twins born alive^1, 2^. Success of separation surgery is affected by many factors including the clinicians experience and an accurate understanding of the anatomy of the affected organs^7, 8^. In order to reduce surgical uncertainty and increase the success rate, it is important to acquire detailed anatomic information and carry out accurate surgical planning prior to separation surgery.

Clinically, various medical imaging techniques and simple medical models are used to support surgical planning before surgical separation of conjoined twins. Typically, anatomic details of conjoined twins are obtained by conventional medical imaging modalities^8–10^. For example, ultrasonography is used to assess the brain^11^; fluoroscopy is used to obtain the anatomic features of anal fistula^10^; and high-resolution computed tomography (CT) and magnetic resonance imaging (MRI) are used to reveal the geometric and positional details of the internal organs^12, 13^. These imaging tools have been used to support successful surgical planning^2, 7, 14, 15^.

In addition to these imaging tools, conjoined twin models made of foam core and silicone rubber have also been used to guide separation surgery^16^. However, in most cases, the phantoms fabricated using traditional methods are restricted by inability to simulate the multidimensionality of the body, with no appropriate fabrication method for reproducing three-dimensional (3D) shape.

More recently, 3D printing can be used to produce the models of conjoined twins for surgical planning. 3D printing represents a family of additive manufacturing processes that are capable of producing complex geometric structures through precise layer-by-layer deposition of materials^17, 18^. Owing to the flexibility for producing freeform geometric features, 3D printing has been widely used in many medical applications, such as medical education, dental customization, skull defect repair and cardiac bypass surgical planning^19–24^.

In this paper, we present a new method that combines 3D printing, assembling, and casting to produce anatomic models of high fidelity for surgical planning of conjoined twin separation. The related anatomic features of the conjoined twins were captured by CT and classified into the following five groups: skeleton group, spinal nerve group, colon group, kidney-bladder group, and other tissue group. The geometric and positional features of these groups were reconstructed from a 3D computer model ^25^. The casting and the assembling processes were designed based on the reconstructed computer models of these organ groups. With acrylonitrile-butadiene-styrene (ABS), silica gel, contrast agents and plasticine, individual internal phantoms were fabricated using a freeform method that combines 3D printing and casting. The produced phantoms were then assembled, positioned firmly within a 3D printed shell mold system simulating the skin boundary, and casted with transparent silica gel. The produced conjoined twin phantom was further scanned by CT for fidelity evaluation. Subsequent data analysis showed that the produced phantom had an overall mean deviation of less than 2 mm in comparison with the original models, closely reassembling the anatomic features of the conjoined twins. These results support the technical potential of simulating structural, optical and CT properties of relevance in conjoined twins.

## Results

### A conjoined twin model produced by casting individually fabricated phantoms

**Figure 1:**
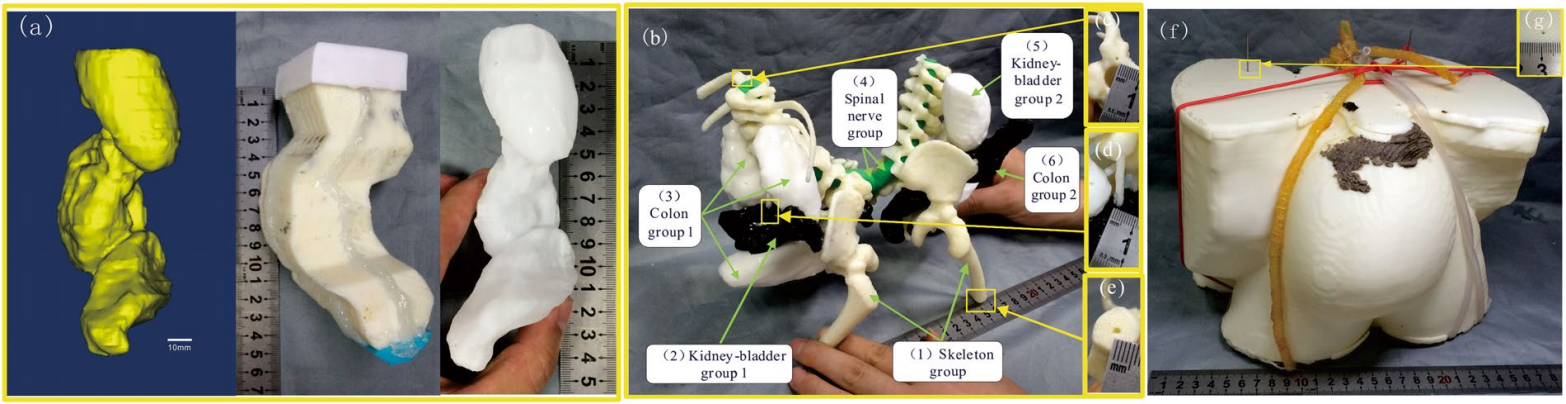
Illustration of the fabrication process for a conjoined twin model: (**a**) Fabrication of a soft tissue phantom (using the kidney-bladder group as an example). From left to right: computer aided design (CAD) model of the kidney-bladder group, shell mold of the kidney-bladder group produced by the FDM process, silica phantom of the kidney-bladder group cast by the shell mold. (**b**) Assembling the phantoms of the five organ groups. (**c**–**e**) Multiple positioning holes and columns are designed on the phantoms to ensure accurate positioning of the phantom assembly. (**f**) The organ assembly is placed inside a shell mold that simulates the body surface for further casting. The position accuracy. (**g**) Positioning hole is designed on the shell mold to ensure accurate positioning of the phantom assembly.

Fabrication of the conjoined twin model was based on casting the phantoms of individual organ groups (Fig. 1a), assembling these phantoms in an assembly (Fig. 1b), and placing the assembly in a shell mold for further casting (Fig. 1c). To fabricate the phantom of each organ group, a shell mold was first produced by the FDM process using the CT data. In order to simulate the visual and CT contrasts of the actual tissue components, different contrast agents were added in silica gel. To produce organ groups 2 and 6, nanoscale tantalum at concentrations of 0.024 g/ml and 0.04 g/ml was added in silica gel respectively to simulate different CT contrasts. To produce organ groups 3 and 5, iomeprol at concentrations of 0.02 ml/ml and 0.04 ml/ml was added respectively. To produce organ group 4, green plasticine was used. To produce organ group 1, the hard skeleton was directly printed by the FDM process using acrylonitrile butadiene styrene (ABS). For the casting of the soft tissue phantoms, the inner surface of the shell molds were costed with a thin layer of release agent to facilitate demolding. The two parts of the shell mold were sealed, filled with silica gel, and placed in a sonication chamber for 5 hrs to prevent precipitation of contrast agents. After 72 hrs, the fully cured phantoms were demolded and assembled. To ensure the positioning accuracy of the assembly, multiple positioning holes and columns were designed on the phantoms (Fig. 1b–g). To produce the final conjoined twin model, a shell mold as shown in Fig. 1f was printed in advance. The thickness of the shell mold was 2 mm to ensure structural strength and stability. As the organ group assembly was placed inside the shell mold, the positioning holes on the assembly and on the shell molds were aligned in order to ensure the positioning accuracy of the organ groups. Transparent silica gel was mixed with the curing agent at a ratio of 10:1 (V/V) and poured into the shell mold to produce the green part. After the green part was cured for 120 hrs at room temperature, it was demolded. The redundant fixtures, the supporting surfaces (both top and bottom), the remaining pieces of the shell mold, and the protective layer were subsequently removed to obtain the final conjoined twin model.

### Simulating visual and CT contrasts of individual organs in a high fidelity fabricated conjoined twin model

**Figure 2:**
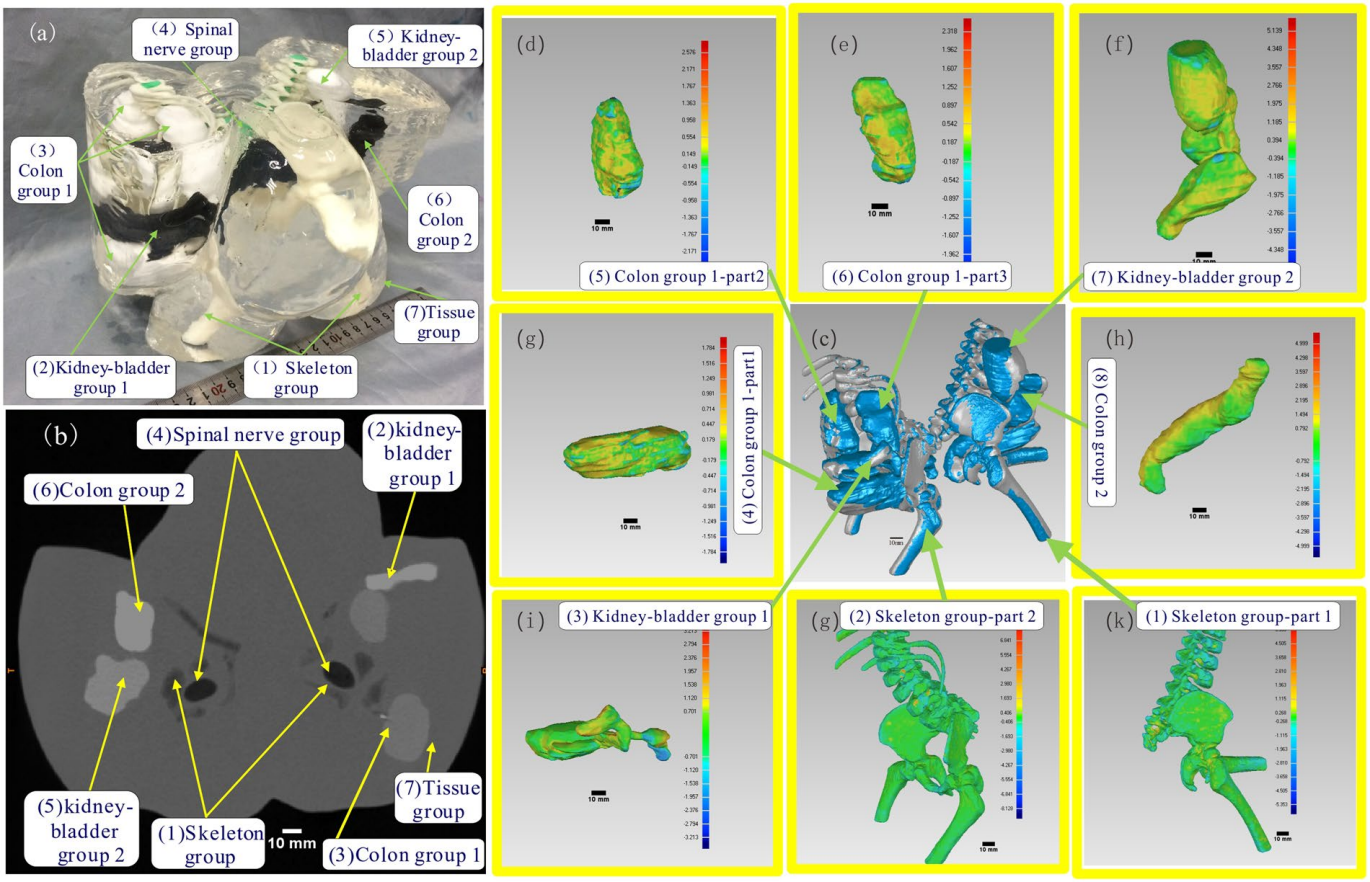
Visual, CT contrasts and fidelity maps of the produced conjoined twin model: (**a**) Isometric view of the conjoined twin model where the internal organs can be clearly differentiated. (**b**) The CT scan image of the conjoined twin model where different organ groups can be clearly delineated. (**c**) Applied iterative closest point algorithm-based alignment 3D CAD models of the organ group reconstructed from the CT data and the actually produced phantom. (**d**–**k**) Collection of the fidelity maps between individual phantoms and their original CT data.

The produced conjoined twin model enable visual delineation of different organ groups, and resemble CT contrasts of biologic tissue, as shown in Fig. 2a,b. The model was subsequently examined by a 64-slice CT (Light Speed VCT, GE Healthcare) at the First Affiliated Hospital of Anhui Medical University. Figure 2b shows a cross-sectional CT image of the conjoined twin model. According to the figure, the averaged CT intensities for individual organ groups are −256, 710, 350, −600, 590, 1050, and 85 HU, respectively. Through both visualization and CT scan, the internal organ groups can be clearly distinguished, indicating the clinical utility of using such a model to guide the surgical planning.

To ensure accurate guidance of the surgical procedure, the produced conjoined twin model should resemble the patient anatomy with fidelity. We have defined a quantitative method for fidelity evaluation based on an iterative closest point (ICP) algorithm. Figure 2d shows the fidelity maps of individual organ group phantoms in comparison with their original CT data, in which the deviation chromatography on each fidelity map can help to analyze error at the exact location. Considering that both the fidelity of individual organ group phantoms and the relative positions of these phantoms in the assembly contribute to the overall fidelity of the conjoined twin model, we have also calculated the fidelity of individual phantoms within the assembly. Table 1 lists the statistics of fidelity maps (Fig. 2d) of individual phantoms before and after assembly. No fidelity was calculated for the spinal nerve group as it was hand-made. As shown from the Table 1, the mean fidelity error of any individual phantom was less than 0.5 mm before assembly and less than 1.5 mm after assembly.

**Table 1:** Errors of the individual phantoms before and after assembling.

Geometry		Mean Error (mm)	Upper Deviation (mm)	Lower Deviation (mm)
Individual phantom	(1) Skeleton group-part 1	0.57	0.74	−0.29
	(2) Skeleton group-part 2	−0.16	0.22	−0.33
	(3) Kidney-bladder group 1	−0.29	1.12	−1.23
	(4) Colon group 1-part 1	−0.45	0.24	−0.64
	(5) Colon group 1-part 2	−0.41	0.29	−0.64
	(6) Colon group 1-part 3	−0.37	1.01	−1.10
	(7) Kidney-bladder group 2	−0.02	1.24	−1.15
	(8) Colon group 2	−0.48	0.51	−0.85
Individual phantom in the assemblies	(1) Skeleton group-part 1	−0.08	0.36	−0.45
	(2) Skeleton group-part 2	−0.16	0.22	−0.33
	(3) Kidney-bladder group 1	0.71	0.94	−0.24
	(4) Colon group 1-part 1	0.21	0.73	−0.49
	(5) Colon group 1-part 2	0.18	0.73	−0.60
	(6) Colon group 1-part 3	0.18	0.46	−0.22
	(7) Kidney-bladder group 2	0.40	0.56	−0.28
	(8) Colon group 2	0.78	0.96	−2.25

## Discussion

Separation surgery for conjoined twins is always challenging because the sharing of organs in each of them is unique^8^. Regardless of the low survival rate of conjoined twins, many of those born alive will require separation surgery in order to live a normal life or just to survive. The feasibility of separation surgery after birth needs to be assessed because the distribution of organs is entirely different in each case^26^. Various imaging techniques provide abundant anatomic information conducive to the assessment of conjoined twins, and the anatomical information obtained from the imaging is of great help in the planning the operative pathway^8, 14^. However, these imaging tools are not interactive and cannot reflect complex 3D information of the tissues. Abdominal ultrasound, plain radiography, and 1D fluoroscopic examinations may provide minimal information. By contrast, advanced computed tomography (CT) and Magnetic Resonance Imaging (MRI) scanning can provide detailed 3D information, but resolution limitation and virtualization cannot provide maximal information^10^. This paper reports a freeform fabrication method for fabricating tissue-simulating phantoms by combining 3D printing and casting. The phantoms simulate not only the structural features of conjoined twins but also CT contrasts between different organs. Moreover, the phantom also allows clinicians to visually distinguish detailed cross information about the organs. Most notably, an ICP algorithm-based fidelity calculation algorithm for 3D shape has been developed. The algorithm offers an understanding of the phantom fabrication quality in terms of shape and position, and represents a possible direction in 3D shape similarity measurement for other applications. The comprehensive value of the conjoined twin model depends on the fabrication quality and assembling fidelity of the individual parts. Compared with the replicable fabrication of phantoms with the same contrast difference, the fidelity of the conjoined twin model is affected by the independent fabrication processes for producing individual organ group phantoms with distinct contrast differences. The primary factors contributing to the fidelity of the final conjoined twin model include the FDM fabrication of the protective layer, the FDM fabrication of the shell molds, and the assembly of individual organ group phantoms. The secondary factors contributing to the overall fidelity include the reconstruction of the solid model, the accuracy of CT scan, and the geometric deformation of silica gel during the curing process. In order to facilitate easy assembly of individual phantoms with high fidelity, the design of the positioning structure should consider the following requirements: (1) the positioning columns must remain perpendicular to the contact surface of the phantoms; (2) three positioning columns are required for each phantom; (3) the length of the positioning columns should be sufficient in order to support secure fitting with the matching holes in the neighboring phantoms; and (4) the center of each positioning hole should coincide with that of the corresponding positioning column. To evaluate the fidelity error caused by the modeling algorithm, we used different people to generate the CAD model of the phantoms and the resultant maximum arithmetic average error in repetitive model reconstruction was 0.35 mm with a deviation of less than 0.4 mm. In addition, the pixel/slice thickness of the CT was 0.825/0.5 mm, and the diameter of the 3D printing nozzle of the FDM printer was 0.25 mm. Further systematic analysis is needed to understand the error sources and their contributions to the fidelity of the final model.

## Conclusion

We have demonstrated the feasibility of producing a high fidelity conjoined twin model with both visual and CT contrasts by combining 3D printing and casting. In the tissue classification procedure, the related organs were divided into five organ groups, with the CAD models reconstructed based on the CT scanning data. Among the five organ groups, the skeleton group was produced by the FDM printer using ABS. The rest four groups of soft tissue were produced by casting silica gel in FDM-produced shell molds. The produced organ group phantoms were assembled, positioned firmly within a shell mold, and cast with transparent silica gel to produce the conjoined twin model. In order to introduce visual and CT contrasts for different organ groups in the model, we have explored a variety of available contrast agents and added them in silica gel for different organ groups. We have also carried out the fidelity analysis for the produced organ group phantoms in comparison with the original CT data. Our study showed that the produced model reassembled the geometric features of the patient with an overall mean deviation of less than 2 mm, indicating the clinical potential to use this method for surgical planning in conjoined twin separation surgery. Consequently, the produced model could be applied in acquiring organ distribution information of the conjoined twin. Whereas the preliminary investigations conducted in this study focused on macro structures in this biological system, phantoms with more complex microstructures can be investigated in future studies.

## Materials and Methods

### Methodology for phantom design and fabrication

In the case of conjoined twin separation surgery, it is likely that improved understanding of organ distribution leads to higher chances of success. Therefore, it is of great significance to fabricate multifunctional phantoms to help surgeons better understand anatomic information.

**Figure 3:**
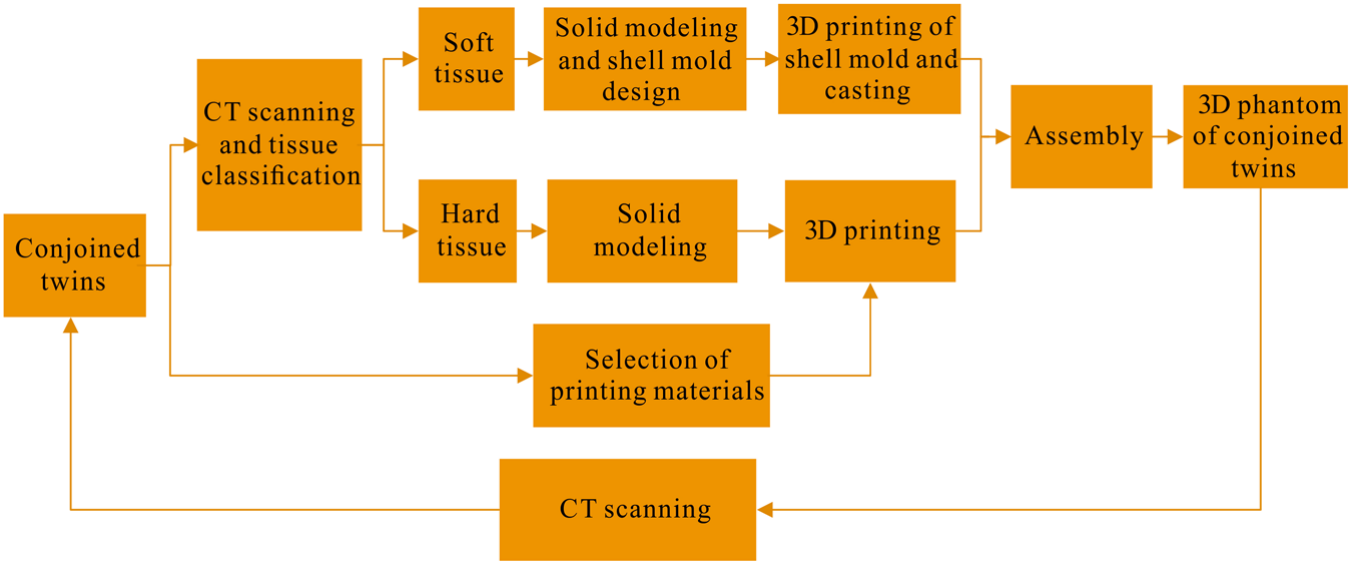
Schematic illustration of the processes involved in fabrication of a conjoined twin model.

The conjoined twin model was fabricated following a cascade of processes as illustrated in Fig. 3. Considering that the routinely used CT scan has a positional accuracy as high as 0.3 mm, the CT scan data were used as the basis for assessing the organ distribution of the conjoined parts. Taking into account the differences in surgical requirements and the internal organs of different interests involved in surgical procedures, organs should be simulated at different accuracy and spatial resolution levels. In this specific surgical case, we divided the internal organs into the following five groups: skeleton, spinal nerve, colon, kidney-bladder, and other tissue. Considering the possibility for future surgical simulation, these organ groups were further divided into hard and soft types based on their mechanical characteristics. The hard phantoms were directly printed by the FDM process, while the soft phantoms were produced by casting silica gel in a 3D printed shell mold based on the CT data. The individual organ group phantoms were then assembled and casted in a shell mold to produce the final conjoined twin model.

### Materials

The contrast enhancement pigments were added in transparent silica gel to simulate both visual and CT contrasts of individual organ groups. For visual differentiation of different organ groups, dyes of different colors were added. To simulate the contrast differences between individual organ groups, positive and negative CT contrast agents at different concentration levels were added. The CT contrast is defined as the X-ray attenuation difference in tissue as Hounsfield units (HU) in CT scanning slices. It works with the visual contrast to provide quantitative boundary information for tissue localization and differentiation in a surgical procedure. On the other hand, the conjoined twin model with organ groups of different contrasts enables subsequently quantitative evaluation of the structural fidelity in comparison with the original CT data. To facilitate quantitative guidance for separation surgery, the phantoms should simulate geometric features of individual organ groups with strong visual contrasts and large differences in the HU values^27, 28^. To facilitate easy visualization of the internal organ structures, the organ groups should be embedded in a transparent phantom material.

**Figure 4:**
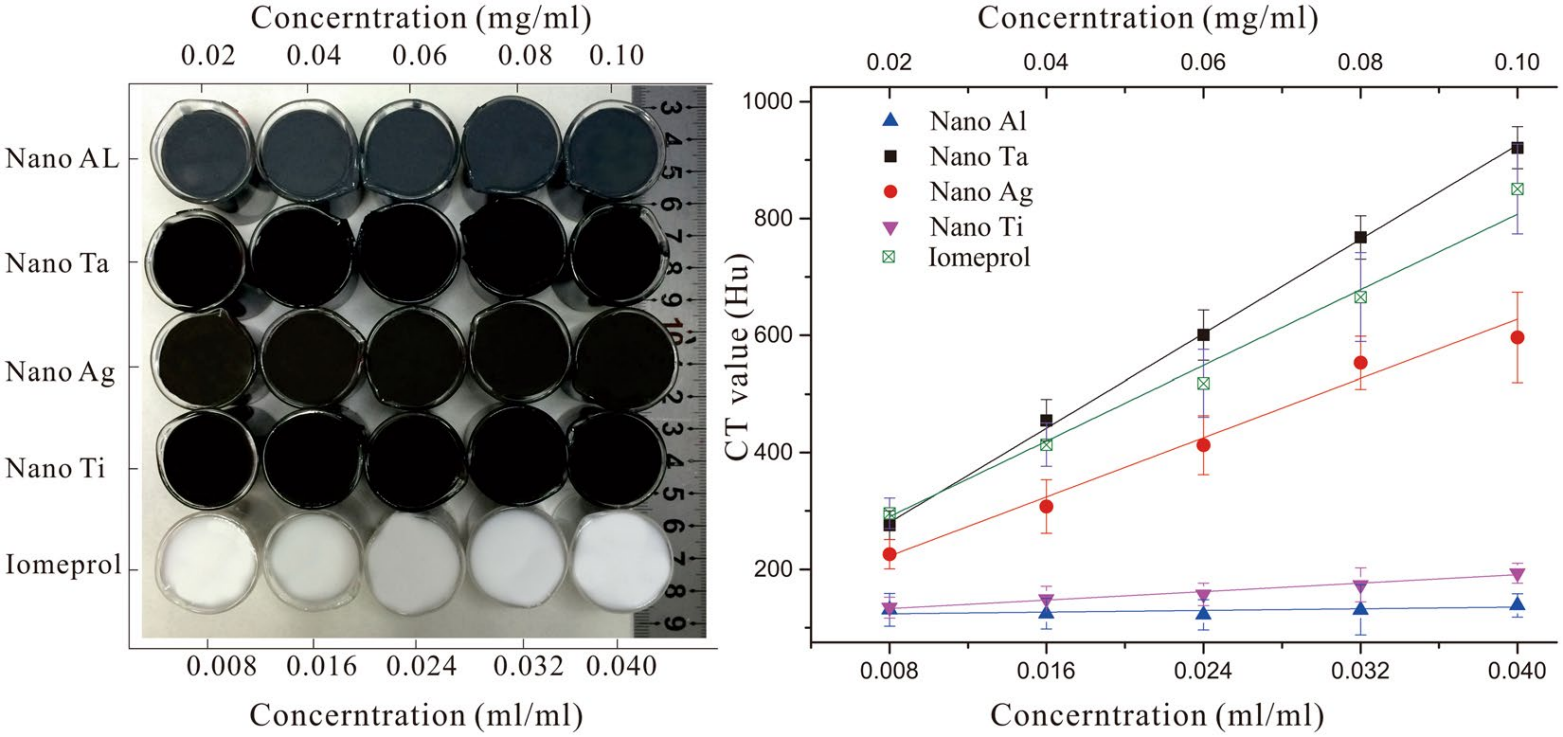
Silica gel phantoms with different types and concentrations of contrast enhancement pigments to simulate different visual and CT contrasts: (**a**) Silica gel mixed with a variety of CT contrast agents at different concentration levels; and (**b**) Quantitative evaluation of the CT contrasts for different contrast agents at different concentration levels.

Transparent silica gel, contrast agents with different colors, ABS and plasticine were chosen as the construction materials for the organ group phantoms. The transparent silica gel (Nanjing Hua Cheng Chemical Co., Ltd, Nanjing, China) is transparent, non-toxic, chemically stable, and with mechanical properties tunable by changing the mixing ratio between the curing agent and the silica gel, making it an ideal base material for constructing tissue-simulating phantoms^29, 30^. To simulate both visual and CT contrasts of the individual organ groups, contrast agents with different colors and different Hu levels were added in the silica gel^27, 28^. Figure 4a shows the silica gel samples cast in small beakers after mixing with nanoscale aluminum, nanoscale tantalum, nanoscale silver, and nanoscale titanium respectively at concentrations of 0.008, 0.016, 0.024, 0.032, and 0.04 g/ml. Similarly, silica gel samples with iomeprol concentrations of 0.02, 0.04, 0.06, 0.08, and 0.10 ml/ml were also produced. After casting, the samples were placed in a sonication chamber for curing in order to prevent precipitation of contrast enhancing pigments. The produced phantoms were subject to a 64-slice CT scan (Light Speed VCT, GE Healthcare) for Hu contrast evaluation, as plotted in Fig. 4b. The casting experiments were triplicated for statistic analysis. According to the figure, we speculated that the CT contrast of the phantom was linearly correlated with the concentration level of the contrast agent within a certain range. In order to achieve simultaneous visual and CT contrasts, we simulated the colon organ group by mixing silica gel with 0.024 and 0.04 g/ml nanoscale tantalum (Shanghai Shui Tian Material Technology Co., Ltd., China); simulating the kidney-bladder organ group by mixing silica gel with 0.02 and 0.04 ml/ml iomeprol (Bracco Imaging Italia SRL, Italia)^31, 32^; simulating the solid skeleton group using light yellow ABS with HU CT value of −333; and simulating the spinal nerve group using a green plasticine with a HU CT value of −750^25^.

### Design and 3D printing of the shell molds for organ groups

**Figure 5:**
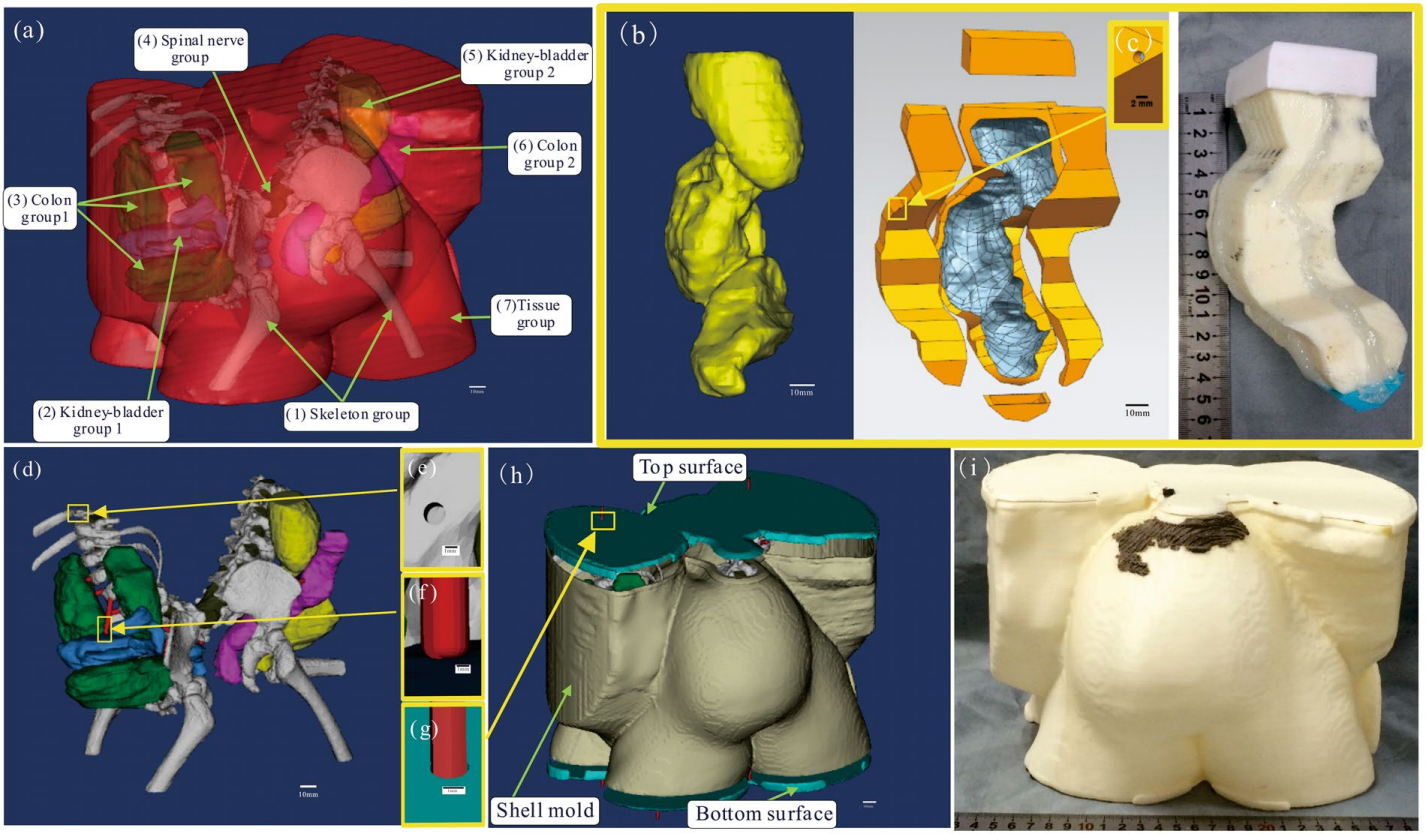
Illustration of the reconstruction of organ computer aided design (CAD) models, design of positioning project for phantom assembling, and fabrication process of molds for soft phantom casting: (**a**) Reconstructed organ CAD models of the conjoined twins. (**b**) Fabrication of a mold for soft tissue phantom casting (using the kidney-bladder group as an example). From left to right: CAD model of the kidney-bladder group, mold system of the kidney-bladder group designed with the aid of software, mold system of the kidney-bladder group produced by the FDM process. (**c**) Hole is designed on mold for marking confection point with positioning column on soft phantom. (**d**) Positioning project design for phantom assembling. (**e**–**g**) Positioning holes and column are designed for assembling individual phantoms. (**h**) The CAD models of shell mold system with organ assembly inside. (**i**) The shell mold system printed by the FDM process with ABS.

The models (Fig. 5a) of the organ group were reconstructed based on the conjoined twins computed tomography (CT) data with the help of the Mimics software package (Mimics 16; Materialise Dental)^33^. They were subsequently exported into editable initial graphics exchange specification format files^23, 34^. Unlike the hard phantoms were printed directly, soft phantoms were casted with the aid of the molds designed using Unigraphics NX package (UG 9.0, Unigraphic Solutions Inc., USA) and Geomagic software package (GeoMagic Studio 2012; GeoMagic USA). Just as Fig. 5b shows, by employing operations such as shell construction etc., molds for inter soft tissue phantom casting were designed respectively. Each of them was divided into 2–4 parts for later demolding^35^. However, the successful fabrication of individual phantoms is not enough, the accuracy of the conjoined twin model also depends on the assembly precision, an appropriate positioning project ensuring assembly precision were thereby expected. Mathematical theory demonstrates that three points in different planes position a rigid object in three dimensional (3D) space. Figure 5d–f show that three positioning columns or holes were designed for each inter individual phantom with the base of hard skeletons. Particularly, the positioning structures were designed to exactly reach the surface of the connected organ, and groove-like structures (Fig. 5f) were helpful in assembling work. Moreover, 2 mm-thick molds (Fig. 5h) for tissue phantom casting were finally designed, and positioning holes (as shown in Fig. 5g) on top and bottom surfaces were coaxial with that on skeleton phantoms, providing precise position of inter individual phantoms inside tissue phantom. All the molds were printed by FDM process. However, limited by the accuracy of the FDM printer, internal surfaces of molds were grainy, which would result in the surface defect of casted phantoms, hence a 0.21 mm-thick smooth protective silica gel layer was fabricated on internal surface of each mold. As a result, all the molds were printed 1.01 times of the original size with a 3D printer (Dimension 1200es, Stratasys, US) to balance the shrinking caused by the protective layer.

### Fidelity calculation based on iterative closest point (ICP) algorithm

To facilitate accurate simulation of tissue anatomy by the 3D printed model, it is critically important to develop a quantitative method for fidelity assessment. Based on the Merriam-Webster Dictionary, fidelity refers to the degree to which something matches or copies something else^14^. For many years, fidelity research has been a topic for better understanding of the human visual perception system^36, 37^. Shape fidelity-based retrieval of 3D data has been a focus of research in various disciplines, such as computer vision^34^, mechanical engineering^38^, artifact searching^39^, molecular biology^35^, and chemistry^33^. The shape fidelity-based retrieval method has been used in numerous applications for model similarity calculation. However, none of these applications are similar to that of the conjoined twin model. We propose a method for fidelity assessment that involves similarity analysis for both the shape and the position of the produced models.

**Figure 6:**
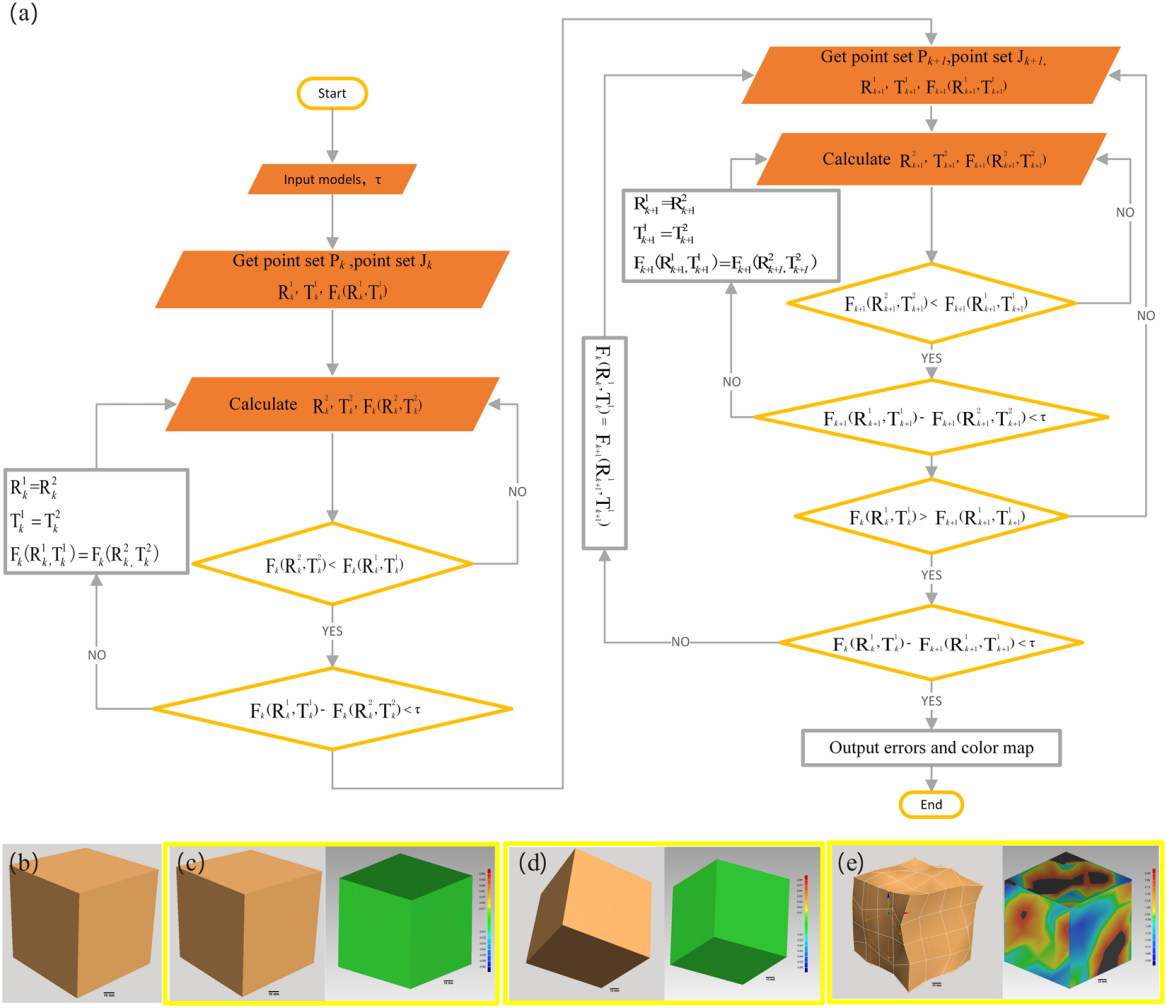
Definition and verification of the fidelity assessment method based on an ICP algorithm: (**a**) Flowchart displaying the processes for fidelity calculation. (**b**) Standard cube model. (**c**–**e**) Random deformations that applied random movement, random rotation, random shape deformation, and color maps got from the ICP algorithm-based fidelity calculation algorithm.

Theoretically, the term fidelity as described in this paper is defined as the averaged error between the reference vertices in the original model and the duplicated model after optimal alignment. Practically, it is difficult to obtain optimal alignment between two models, not only because the corresponding models are unordered and without a unified world coordinate, but also because both models in this research were in STL format with numerous vertices. To calculate the fidelity parameters and the corresponding color map fo an organ group phantom, we developed a procedure as shown by the flowchart in Fig. 6a. This procedure was further validated by a Geomagic software package (GeoMagic Studio 2012; GeoMagic USA)^40^.

Before fidelity is calculated, the models were first aligned in a unified coordinate system using an ICP algorithm^41, 42^. As shown in the flowchart (Fig. 6a), the ICP alignment is implemented by calculating the best suitable rotation matrix and translation matrix. Takes the k-th alignment for example. *τ* is the tolerance. P_*k*_ = {${{\rm{p}}}_{k}^{i}$} and Z_*k*_ = {${{\rm{z}}}_{k}^{i}$} (*i* = 1, 2 … N) are stochastic point sets of corresponding models, where N are the number of the stochastic point, and point ${{\rm{p}}}_{k}^{i}$ corresponds to point ${{\rm{z}}}_{k}^{i}$. ${{\rm{R}}}_{k}^{j}$ and ${{\rm{T}}}_{k}^{j}$(*j* = 1, 2) are rotation matrix and translation matrix respectively. F_*k*_(${{\rm{R}}}_{k}^{j}$, ${{\rm{T}}}_{k}^{j}$) (*j* = 1, 2) is standard least-squares distance between point sets.

According to the best fitting principle, there should be an ideal rotation matrix ${{\rm{R}}}_{k}^{j}$ and translation matrix ${{\rm{T}}}_{k}^{j}$, so that the set ${{\rm{R}}}_{k}^{j}$P_*k*_ + ${{\rm{T}}}_{k}^{j}$ aligns best with Z_*k*_^42^, and the least-squares distance function (1) is minimized.

1 $${{\rm{F}}}_{k}({{\rm{R}}}_{k}^{j},{{\rm{T}}}_{k}^{j})=\sum _{i=1}^{{\rm{N}}}\,{|{{\rm{p}}}_{k}^{i}{{\rm{R}}}_{k}^{j}+{{\rm{T}}}_{k}^{j}-{z}_{k}^{i}|}^{2}$$

Calculation of ideal rotation matrix ${{\rm{R}}}_{k}^{j}$ and translation matrix ${{\rm{T}}}_{k}^{j}$ begins from the initialization of ${{\rm{R}}}_{k}^{1}$ and ${{\rm{T}}}_{k}^{1}$. Lets C_*p*_ in (2) be the center of the point set P_*k*_, and C_*z*_ in (3) to be the center of the point set Z_*K*_.

2 $${{\rm{C}}}_{p}=\sum _{i=1}^{{\rm{N}}}\,{{\rm{p}}}_{k}^{i}$$

3 $${{\rm{C}}}_{z}=\sum _{i=1}^{{\rm{N}}}\,{{\rm{z}}}_{k}^{i}$$

As for the nest rotation matrix ${{\rm{R}}}_{k}^{2}$ and translation matrix ${{\rm{T}}}_{k}^{2}$, ${{\rm{T}}}_{k}^{2}$ = C_*z*_ − ${{\rm{R}}}_{k}^{2}$C_*p*_.

Lets ${\tilde{{\rm{P}}}}_{k}^{i}={{\rm{p}}}_{k}^{i}-{{\rm{C}}}_{p}$, ${\tilde{{\rm{Z}}}}_{k}^{i}={{\rm{z}}}_{k}^{i}-{{\rm{C}}}_{z}$. To compute the next rotation ${{\rm{R}}}_{k}^{2}$, the problem is equivalently simplified into maximizing the one-variable objective function (4).

4 $${{\rm{F}}}_{K}({\rm{R}})=\sum _{i=1}^{{\rm{N}}}\,\langle {\tilde{{\rm{P}}}}_{k}^{i}{\rm{R}},{\tilde{{\rm{Z}}}}_{k}^{i}\rangle $$

Let ${\rm{H}}={\sum }_{i=1}^{{\rm{N}}}\,{\tilde{{\rm{P}}}}_{k}^{i}\,{({{\rm{Z}}}_{k}^{i})}^{T}$, which is a 3 × 3 matrix. According to both orthogonal property of rotation matrix and singular value decomposition principle^41, 43^, there should be 3 × 3 orthogonal matrices, for example U and V, and 3 × 3 diagonal matrix Λ with non-negative elements exist such that H = UΛV. According to the singular value decomposition (SVD) algorithm in the least-squares method^41^, to maximize the function F_*K*_(R), the next rotation matrices are given by (5).

5 $${{\rm{R}}}_{k}^{2}=\{\begin{array}{cc}{{\rm{V}}{\rm{U}}}^{T} & det\,({{\rm{V}}{\rm{U}}}^{T})=1\\ {\rm{V}}\,(\begin{array}{ccc}1 & 0 & 0\\ 0 & 1 & 0\\ 0 & 0 & -1\end{array})\,{{\rm{U}}}^{T} & det\,({{\rm{V}}{\rm{U}}}^{T})=-1\end{array}$$

The rotation matrices ${{\rm{R}}}_{k}^{1}$, ${{\rm{R}}}_{k}^{1}$ and the translation matrices ${{\rm{T}}}_{k}^{1}$, ${{\rm{T}}}_{k}^{2}$ that satisfy both function (6) and function (7) are calculated and saved.

6 $${{\rm{F}}}_{k}({{\rm{R}}}_{k}^{{\rm{2}}},{{\rm{T}}}_{k}^{{\rm{2}}}) < {{\rm{F}}}_{k}({{\rm{R}}}_{k}^{{\rm{1}}},{{\rm{T}}}_{k}^{{\rm{1}}})$$

7 $${{\rm{F}}}_{k}({{\rm{R}}}_{k}^{{\rm{1}}},{{\rm{T}}}_{k}^{{\rm{1}}})-{{\rm{F}}}_{k}({{\rm{R}}}_{k}^{{\rm{2}}},{{\rm{T}}}_{k}^{{\rm{2}}}) < \tau $$

Then, the new stochastic point sets P_*k*+1_ and Z_*k*+1_ are chosen, and the corresponding least distance F_*k*+1_(${{\rm{R}}}_{k+1}^{2}$, ${{\rm{T}}}_{k+1}^{2}$) is calculated. The calculation iterates until both function (8) and function (9) are satisfied. Finally, the errors are the calculated and the fidelity maps are generated.

8 $${{\rm{F}}}_{k+{\rm{1}}}({{\rm{R}}}_{k+{\rm{1}}}^{{\rm{1}}},{{\rm{T}}}_{k+{\rm{1}}}^{{\rm{1}}}) < {{\rm{F}}}_{k}({{\rm{R}}}_{k}^{{\rm{1}}},{{\rm{T}}}_{k}^{{\rm{1}}})$$

9 $${{\rm{F}}}_{k}({{\rm{R}}}_{k}^{{\rm{1}}},{{\rm{T}}}_{k}^{{\rm{1}}})-{{\rm{F}}}_{k+{\rm{1}}}({{\rm{R}}}_{k+{\rm{1}}}^{{\rm{1}}},{{\rm{T}}}_{k+{\rm{1}}}^{{\rm{1}}}) < \tau $$

Models of various complexities and their random deformations are used to validate the feasibility of the aforementioned fidelity calculation algorithm. To he shape deformed models were obtained by the free-form deformation (FFD) technique that deforms an object enclosed within a cube by moving the control points on its edges^44^. Thedeformation of the model is defined by a matrix ﻿random (M, R, V), where ﻿three independent variables M﻿, R, and V represent translation, rotation, and distortion, respectively. The default value of M (or R) is 0 and it changes to ﻿1 as the deformation involves random translation (or rot﻿ation). The value of V (ranging from 0 to 1) is defined as proportion of deformed control points in the FFD model. For validation of the fidelity calculation algorithm in solid models with moderate deformation, the random motion of the control points in the FFD model is restricted within 10% of the longest axis. As an example, fidelity analysis is carried out in a standard cube model (Fig. 6b), and the resultant fidelity maps after translation, rotation, and distortion operations are shown in Figs. 6c, 6d, and 6e, respectively. According to the fidelity maps, neither translation nor rotation affect the fidelity of the solid model, while distortion induces the distributed fideltiy changes. Table 2 lists the fidelity analysis results for 3D models of various geometrical feastures and deformations. The results in Table ﻿2 indicate that neither random translation nor rotation induce any fidelty issue in a solid model, while distortion induces fidelity errors proportional to the positional deviation of the control points. It concludes that the ICP -based fidelity calculation algorithm is an effective method to assess the similarity between solid models in the case of moderate deformation.

**Table 2:** Fidelity analysis for various geometric features and deformations.

Geometry	Deformation operation	Fidelity calculation			Standard Deviation
		Mean error (mm)	Upper error (mm)	Lower error (mm)	
	random (1, 0, 0)	0	0	0	0
	random (1, 1, 0)	0	0	0	0
	random (1, 0, 0.1)	−0.03	3.18	−3.69	0.56
	random (0, 1, 0)	0	0	0	0
	random (1, 1, 0.5)	−0.13	5.33	−4.06	1.16
	random (0, 1, 0.7)	−28	7.84	−8.09	1.36
	random (1, 0, 0)	0	0	0	0
	random (1, 1, 0)	0	0	0	0
	random (1, 0, 0.2)	0.55	10	−2.9	1.75
	random (0, 1, 0)	0	0	0	0
	random (1, 1, 0.5)	0.31	9.48	−10.12	2.65
	random (0, 1, 0.8)	1.91	10.23	−7.02	3
	random (1, 0, 0)	0	0	0	0
	random (1, 1, 0)	0	0	0	0
	random (1, 0, 0.1)	0.35	3.19	−4.23	0.61
	random (0, 1, 0)	0	0	0	0
	random (1, 1, 0.3)	0.08	3.26	−3.26	0.86
	random (0, 1, 0.7)	−0.12	3.75	−4.38	1.16
	random (1, 0, 0)	0	0	0	0
	random (1, 1, 0)	0	0	0	0
	random (1, 0, 0.3)	−0.61	0.68	−3.14	0.81
	random (0, 1, 0)	0	0	0	0
	random (1, 1, 0.5)	−0.02	1.77	−1.56	0.59
	random (0, 1, 0.7)	−0.1	2.35	−2.98	0.77
	random (1, 0, 0)	−0.29	5.27	−4.21	0.59
	random (1, 1, 0)	0	0	0	0
	random (1, 0, 0.1)	0.19	7.17	−7.61	1.19
	random (0, 1, 0)	0	0	0	0
	random (0, 1, 0.3)	−0.21	8.19	−8.89	1.86
	random (1, 1, 0.5)	0.63	8.19	−8.19	2.23

## References

[1] Chatterjee SK, Debmaulik T, Talukder B, Sen B, Sarangi B (1986). Separation of pygopagus twins. Pediatric surgery international.

[2] Inamdar SA, Goel SS, Subhedar VS (2012). A thoracophagus conjoined twins with myelomeningocele: an unusual case. Int J Reprod Contracept Obstet Gynecol.

[3] Stuart GM, Black AE, Howard RF (2015). The anaesthetic management of conjoined twins. Seminars in Pediatric Surgery.

[4] Spencer R (2000). Theoretical and analytical embryology of conjoined twins: part i: embryogenesis. Clinical Anatomy.

[5] Hall JG (2003). Twinning. The Lancet.

[6] Spitz L (2015). Ethics in the management of conjoined twins. Seminars in Pediatric Surgery.

[7] Awasthi R, Iyengar R, Rege S, Jain N (2015). Surgical management of pygopagus conjoined twins with spinal bifida. European Spine Journal.

[8] Tannuri ACA, Batatinha JAP, Velhote MCP, Tannuri U (2013). Conjoined twins: twenty years’ experience at a reference center in brazil. Clinics.

[9] Kamata M (2016). Case report anesthetic management of pygopagus conjoined twins: techniques to evaluate cross-circulation. Int J Clin Exp Med.

[10] Watson SG, McHugh K (2015). Conjoined twins: Radiological experience. Seminars in Pediatric Surgery.

[11] Petersen S (2014). 3d assisted prenatal sonographic diagnosis of dicephalic conjoined twins and subsequent planned vaginal delivery. Case Reports in Women’s Health.

[12] Saito K (2001). Construction of a computed tomographic phantom for a japanese male adult and dose calculation system. Radiation and environmental biophysics.

[13] Castadot P, Geets X, Lee JA, Christian N, Grégoire V (2010). Assessment by a deformable registration method of the volumetric and positional changes of target volumes and organs at risk in pharyngo-laryngeal tumors treated with concomitant chemo-radiation. Radiotherapy and Oncology.

[14] Rode H (2006). Four decades of conjoined twins at red cross children’s hospital-lessons learned. South African Medical Journal.

[15] Jr O (1988). Surgical experience with thirteen conjoined twins. Annals of surgery.

[16] Rhodes JL, Yacoe M (2013). Preoperative planning for the separation of omphalopagus conjoined twins the role of a multicomponent medical model. Journal of Craniofacial Surgery.

[17] Sachs E, Cima M, Williams P, Brancazio D, Cornie J (1992). Three dimensional printing: rapid tooling and prototypes directly from a cad model. Journal of Engineering for Industry.

[18] Lipson, H. & Kurman, M. *Fabricated*: *The new world of 3D printing* (John Wiley & Sons, 2013).

[19] Butscher A, Bohner M, Hofmann S, Gauckler L, Müller R (2011). Structural and material approaches to bone tissue engineering in powder-based three-dimensional printing. Acta Biomaterialia.

[20] Wu, W., DeConinck, A. & Lewis, J. A. Omnidirectional printing of 3d microvascular networks. *Advanced Materials* **23** (2011). 10.1002/adma.201004625 21438034

[21] Gao Q, He Y, Fu J-Z, Liu A, Ma L (2015). Coaxial nozzle-assisted 3d bioprinting with built-in microchannels for nutrients delivery. Biomaterials.

[22] Murphy SV, Atala A (2014). 3d bioprinting of tissues and organs. Nature biotechnology.

[23] Klammert U (2010). 3d powder printed calcium phosphate implants for reconstruction of cranial and maxillofacial defects. Journal of Cranio-Maxillofacial Surgery.

[24] Wang, H. *et al*. Three-dimensional virtual surgery models for percutaneous coronary intervention (pci) optimization strategies. *Scientific reports* **5**, 10945–10945 (2014). 10.1038/srep10945 PMC4455241 26042609

[25] Shen S (2016). Freeform fabrication of tissue-simulating phantoms by combining three-dimensional printing and casting. Proc. SPIE.

[26] Spitz L, Kiely E (2002). Experience in the management of conjoined twins. British journal of Surgery.

[27] Schneider U, Pedroni E, Lomax A (1996). The calibration of ct hounsfield units for radiotherapy treatment planning. Physics in medicine and biology.

[28] Hebb AO, Poliakov AV (2009). Imaging of deep brain stimulation leads using extended hounsfield unit ct. Stereotactic and functional neurosurgery.

[29] MacFarlane M, Rosen J, Hannaford B, Pellegrini C, Sinanan M (1999). Force-feedback grasper helps restore sense of touch in minimally invasive surgery. Journal of Gastrointestinal Surgery.

[30] Azar FS, Metaxas DN, Miller RT, Schnall MD (2000). Methods for predicting mechanical deformations in the breast during clinical breast biopsy. Methods.

[31] Norrish K, Hutton JT (1969). An accurate x-ray spectrographic method for the analysis of a wide range of geological samples. Geochimica et cosmochimica acta.

[32] Galperin A (2007). Radiopaque iodinated polymeric nanoparticles for x-ray imaging applications. Biomaterials.

[33] Lee K-S, Kim S-H (2010). Non-uniform deformation of an stl model satisfying error criteria. Computer-Aided Design.

[34] Huang S-H, Zhang L-C, Han M (2002). An effective error-tolerance slicing algorithm for stl files. The International Journal of Advanced Manufacturing Technology.

[35] Nee A, Fu M, Fuh J, Lee K, Zhang Y (1998). Automatic determination of 3-d parting lines and surfaces in plastic injection mould design. CIRP Annals-Manufacturing Technology.

[36] Yantis, S. *Visual perception*: *Essential readings* (Psychology Press, 2001).

[37] Fechner, G. *et al*. *Elements of psychophysics* (Holt, Rinehart & Winston, 1965).

[38] Bassoli E, Gatto A, Iuliano L, Grazia Violante M (2007). 3d printing technique applied to rapid casting. Rapid Prototyping Journal.

[39] Lauriks, W. Low density foams. *Low density cellular plastics*: *Physical basis of behaviour* **319** (2012).

[40] Brassard, G. & Salvail, L. Lecture notes in computer science. In *Advances in Cryptology Eurocrypt93*, vol. 765, 410–23 (1994).

[41] Arun, K. S., Huang, T. S. & Blostein, S. D. Least-squares fitting of two 3-d point sets. *IEEE Transactions on pattern analysis and machine intelligence* 698–700 (1987). 10.1109/tpami.1987.4767965 21869429

[42] Ying S, Peng J, Du S, Qiao H (2009). A scale stretch method based on icp for 3d data registration. IEEE Transactions on Automation Science and Engineering.

[43] Yan P, Bowyer KW (2007). A fast algorithm for icp-based 3d shape biometrics. Computer Vision and Image Understanding.

[44] Sederberg TW, Parry SR (1986). Free-form deformation of solid geometric models. ACM SIGGRAPH computer graphics.

